# A Novel RNA-Binding Protein Involves ABA Signaling by Post-transcriptionally Repressing *ABI2*

**DOI:** 10.3389/fpls.2017.00024

**Published:** 2017-01-24

**Authors:** Jianwen Xu, Yihan Chen, Luofeng Qian, Rong Mu, Xi Yuan, Huimin Fang, Xi Huang, Enshun Xu, Hongsheng Zhang, Ji Huang

**Affiliations:** ^1^State Key Laboratory of Crop Genetics and Germplasm Enhancement, Nanjing Agricultural UniversityNanjing, China; ^2^Institute of Industrial Crops, Jiangsu Academy of Agricultural ScienceNanjing, China; ^3^Jiangsu Collaborative Innovation Center for Modern Crop Production, Nanjing Agricultural UniversityNanjing, China

**Keywords:** abiotic stress, ABA signaling, ABI2, *Arabidopsis thaliana*, RNA-binding protein, zinc finger protein

## Abstract

The *Stress Associated RNA-binding protein 1* (*SRP1*) repressed by ABA, salt and cold encodes a C2C2-type zinc finger protein in *Arabidopsis*. The knock-out mutation in *srp1* reduced the sensitivity of seed to ABA and salt stress during germination and post-germinative growth stages. In contrast, *SRP1*-overexpressing seedlings were more sensitive to ABA and salt compared to wild type plants. In the presence of ABA, the transcript levels of ABA signaling and germination-related genes including *ABI3. ABI5. EM1* and *EM6* were less induced in *srp1* compared to WT. Interestingly, expression of *ABI2* encoding a protein phosphatase 2C protein were significantly up-regulated in *srp1* mutants. By *in vitro* analysis, SRP1 was identified as a novel RNA-binding protein directly binding to 3′UTR of *ABI2* mRNA. Moreover, transient expression assay proved the function of SRP1 in reducing the activity of luciferase whose coding sequence was fused with the *ABI2* 3’UTR. Together, it is suggested that SRP1 is involved in the ABA signaling by post-transcriptionally repressing *ABI2* expression in *Arabidopsis*.

## Introduction

Post-transcriptional regulatory events, such as RNA processing, splicing and transport, are crucial to plant growth, development and response to environmental stresses (Ambrosone et al., 2012; Jung et al., 2013). RNA-binding proteins (RBPs) play important roles in post-transcriptional regulation by directly binding to single/double strand RNA (ssRNA/dsRNA) molecules (Ambrosone et al., 2012). RBPs are widely distributed among plant species. More than 200 putative RBP genes have been identified in *Arabidopsis thaliana*, and ∼250 in rice (*Oryza sativa* L.) (Lorković, 2009; Cook et al., 2011; Ambrosone et al., 2012). In the past fifteen years, the researches on the roles of RBPs in the regulation of plant development and stress responses have been gradually increased (Ambrosone et al., 2012; Jung et al., 2013).

RBPs contain several common motifs such as RNA recognition motif (RRM), K homology (KH) domain (Lorković and Barta, 2002), ZnF domain (mainly CCCH type) (Kim et al., 2007; Qu et al., 2014), DEAD/DEAH box (Owttrim, 2006), Pumilio/FBF (PUF) domain, double-stranded RNA-binding domain (DS-RBD) (Tam et al., 2010), Piwi/Argonaute/Zwiklle (PAZ) domain (Song et al., 2003), and the auxiliary domains (glycine-rich, arginine-rich, arginine-glycine, or serine-arginine repeats) (Alba and Pagès, 1998; Ambrosone et al., 2012). The abscisic acid (ABA) is an important plant hormone playing critical roles in many aspects of plant growth and stress responses. Several ABA-regulated RBPs have been reported to be involved in plant stress responses so far (Ambrosone et al., 2012). The RBPs, such as cap binding complex (CBC) (Papp et al., 2004), AAPK interacting protein 1 (AKIP1) (Lamond and Spector, 2003), glycine-rich RNA-binding protein (GRP) (Wang et al., 2012), and ABA-regulated RNA-binding protein (ARP1) (Jung et al., 2013) are all involved in ABA. Cap Binding Protein 20 (CBP20) is the subunit of CBC and the *cbp20* is hypersensitive to ABA (Papp et al., 2004). The recent study has revealed the splicing function of CBP20, and the loss function of it induces the improper splicing of Indeterminate Domain 14 (IDD14) transcription factor and results in the hypersensitivity to salt stress (Kong et al., 2014). RZ-1 is a kind of GRP proteins. RZ-1a, one member of RZ-1 orthologs, is reported to has negative impact on seed germination (Kim et al., 2007). The homologous proteins of RZ-1a, RZ-1b and RZ-1c have been revealed to play critical roles in regulating pre-mRNA splicing (Wu et al., 2015). ARP1 is a putative RBP and modulates several germination responsive genes under ABA (Jung et al., 2013).

Our previous work has identified a novel protein family (SRZ family) with C2C2-type zinc finger motifs from rice (*Oryza sativa* L.) (Huang et al., 2008). The SRZ1 contains three C2C2-type zinc finger motifs (DWXCX_1-4_CX_3_NX_6_CX_2_C, X means any residues), and it is down-regulated by ABA and abiotic stresses (Huang et al., 2008). However precise functions of SRZ proteins remain to be revealed. In present study, we analyzed the function of Stress Associated *RNA-binding protein 1* (*SRP1*), a SRZ1 homolog in *Arabidopsis*, and showed that SRP1 was a novel RNA binding protein involved in the ABA signaling by binding to 3′UTR region of *ABI2* and triggering the instability of *ABI2* mRNA.

## Materials and Methods

### Plant Materials

The *Arabidopsis* Columbia (Col-0) ecotype wild-type (WT), *srp1-1* (SAIL_187_C10), *srp1-2* (SAIL_312_E06), *aba3-1* (CS157), and *35S::SRP1* transgenic lines were used in this work. The mutants were obtained from Arabidopsis Biological Resource Center^1^ (ABRC). *35S::SRP1* transgenic lines were generated by *Agrobacterium*-mediated transformation in this study. The seeds were sown on 1/2 Murashige and Skoog (MS) medium with 3% (w/v) sucrose and 0.8% agar in a light homoeothermic incubator (24 °C) with 16 h light/8 h dark. After 3 days, the germinated seeds were transferred into pot (*R* = 3 cm, *H* = 8 cm) filled with nutritional soil and vermiculite (1:1) and grown under 50 μmol m^-2^ s^-1^ light at 22°C and 16 h light/8 h dark in a greenhouse. The seeds harvested at the same time were used for the experiment.

### Germination and Post-Germination Growth Assays

One hundred seeds each line were sown in the plate (10 cm × 10 cm × 1 cm) with 1/2 MS medium containing different concentrations of ABA or NaCl in triplicates. The plates with seeds were stored at 4°C in dark for four days, and subsequently incubated in the growth chamber (22°C) with 16 h light/8 h dark. Germination and green cotyledon rates were scored at the indicated times after the incubation. Statistical analysis was performed with three biological replicates. Seeds were counted as germinated when the radicle tip had fully penetrated the seed coat (radicle protrusion) as previously described (Liu et al., 2013).

### Generation of SRP1 Transgenic Line and Mutant Identification

The coding sequence of *SRP1* was cloned into binary vector pCAMBIA1300s which was further transferred into *Agrobacterium tumefaciens* strain EHA105. The *Agrobacterium*-mediated transformation in *Arabidopsis* was conducted as previously described (Clough and Bent, 1998). Transgenic plants were screened in hygromycin (25 mg/L)-containing medium and the positive seedlings were randomly selected to be verified by the primers for the hygromycin resistance gene (**Supplementary Table 1**). Mutants *srp1-1* (SAIL_187_C10) and *srp1-2* (SAIL_312_E06) for the *SRP1* gene were identified using the primers LP, RP and pCSA110LB for amplification of T-DNA insertion. The mutants or the transgenic line were further verified by semi-quantitative RT-PCR or qRT-PCR analysis. The primers were listed in **Supplementary Table 1**.

### Real-time PCR

Total RNA were prepared from WT, *srp1* and transgenic lines as previously described (Gutierrez et al., 2009). Approximately 5 μg of RNA were treated with DNase I using a DNA free Kit (Thermo Fisher Scientific, Waltham, MA, USA), and cDNA was synthesized by reverse transcribing total RNA using SuperScript III reverse transcriptase (Invitrogen, Foster City, CA, USA) with oligo (dT)_12_ primer according to the manufacturer’s instructions. Transcript levels of ABA signaling-related genes *RD29A* (AT5G52310), *RD29B* (AT5G52300), *ABI1* (AT4G26080), *ABI2* (AT5G57050), *ABI3* (AT3G24650), *ABI5* (AT2G36270), *EM1* (AT3G51810), and *EM6* (AT2G40170) were assessed by real-time quantitative reverse transcription PCR (qRT-PCR). *Arabidopsis Actin7* was used as the internal control for the qRT-PCR, respectively (Liu et al., 2007; Yelagandula et al., 2014). The primers were listed in **Supplementary Table 1**.

### Subcellular Localization

The transient expression vector was constructed by cloning *SRP1* into pA7-GFP to fuse with GFP. The pA7-GFP vector was used as a control. The vectors were expressed transiently in *Arabidopsis* leaf protoplasts using the PEG (polyethylene glycol) method (Abel and Theologis, 1994). DAPI (4′,6-diamidino-2-phenylindole) was used to dye the nucleus. The visualization of SRP1-GFP or GFP was observed using the Leica SP2 confocal laser scanning microscope.

### RNA Electrophoretic Mobility Shift Assay

The *SRP1* coding sequence was cloned into the expression vector pGEX-2T to generate GST-labeled SRP1 protein. The fusion protein was prepared according to the GST Gene Fusion System Handbook (Amersham Biosciences, Piscataway, NJ, USA). The 3′UTR sequences of *ABI2/3* dsDNA were cloned with the primers containing the T7 promoter sequence (Rohde et al., 2000; Ohta et al., 2003). The 3′UTRs of *ABI2/3* ssRNA was labeled using T7 RNA polymerase and biotin-16 UTP according to the instruction manual (Thermo Fisher Scientific, Waltham, MA, USA). The binding reactions were performed by incubating biotin-labeled RNA with recombinant protein at 23°C for 30 min in 10 μl of binding buffer containing 20 mM 4-(2-hydroxyethyl)-1-piperazineethanesulfonic acid (HEPES) (pH 7.5), 50 mM NaCl, 5 mM 2-mercaptoethanol, 1 mM DL-Dithiothreitol (DTT), 5 μM ZnCl_2_ and 4 mM MgCl_2_ as described (Qu et al., 2014). Electrophoresis was performed at 4°C in 8% native TRIS borate-acrylamide (29:1) gels, which were transferred to N+ nylon membrane (Roche, Basel, Swiss). The membrane was exposed using the Chemiluminescent Biotin-labeled Nucleic Acid Detection Kit (Thermo Fisher Scientific, Waltham, MA, USA), and scanned by ChemiDoc XRS+ imager (Bio-Rad, Irvine, CA, USA). For competition assays, biotin-labeled RNA and recombinant protein were incubated prior to the addition of unlabeled competitor RNA. The sequences of *ABI2* and *ABI3* 3′UTRs were listed in **Supplementary Table 2**.

### Transactivation Activity Assay in Protoplast System

In protoplast system analyzing the effect of SRP1 on the stability of mRNA, the control reporter vector 3× DRE-LUC was constructed by inserting three copies of dehydration response element (DRE, TACCGACAT) and a minimal TATA region of 35S promoter of Cauliflower Mosaic Virus (CaMV) into the upstream of the *firefly luciferase* (*LUC*) in pGL3-basic vector. The reporter vectors 3× DRE-LUC-*ABI2* 3′UTR and 3× DRE-LUC-*ABI3* 3′UTR were constructed by insertion of *ABI2* and *ABI3* 3′UTR into the downstream of luciferase coding sequence. Effector vectors 35S-DREB1A and 35S-SRP1 were constructed by insertion of the *DREB1A* (AT4G25480) or *SRP1* coding region into PA7 vector (Narusaka et al., 2003). The pPTRL (*Renilla reniformis Luciferase* driven by 35S promoter) was used as the internal control. The effectors, reporters and internal control were co-transfected into *Arabidopsis* protoplasts. After culturing for 16 h, the luciferase assays were performed with the Dual-Luciferase Reporter Assay System (Promega, Madison, WI, USA) and quantitated by the GloMax 20/20 luminometer (Promega, Madison, WI, USA). Statistical analysis was performed with three biological replicates.

To analyze the transactivation activity of SRP1, the assay in *Arabidopsis* protoplast system was performed as previously described (Fang et al., 2015). The reporter vector was constructed by inserting four copies of GAL4 binding element and a minimal TATA region of 35S promoter of *CaMV* into the upstream of the *firefly LUC* in pPCV814 vector. The control effector vector 35S-Gal4DB was constructed by inserting the *GAL4DB* into pRTL2-GUS vector. The SRP1 was cloned into control effector vector to generate 35S-Gal4DB-SRP1 as the effector. The pPTRL was used as the internal control. The effectors, reporters and internal control were co-transfected into *Arabidopsis* protoplasts. After culturing for 16 h, the luciferase assays were performed with the Dual-Luciferase Reporter Assay System (Promega, Madison, WI, USA) and quantitated by the GloMax 20/20 luminometer (Promega, Madison, WI, USA). Statistical analysis was performed with three biological replicates.

### Transactivation Activity Assay in Yeast

The transactivation activity assays in the yeast were performed using the Matchmaker GAL4 Two-Hybrid System 3 (Clontech, Mountain View, CA, USA). BD-SRP1 was constructed by cloning the full-length CDS of *SRP1* into pGBKT7 to fuse with the GAL4 binding domain. The construct and the empty vectors were transformed into the yeast strain AH109. The yeast cells were plated onto synthetic dextrose (SD)/-Trp medium to confirm the transformation. At the same time, the yeast cells were plated onto SD/-His/-Trp/-Leu containing 3 mM 3-amino-1,2,4-triazole medium to screen for transactivation activity.

## Results

### Identification and Characterization of SRP1

**FIGURE 1:**
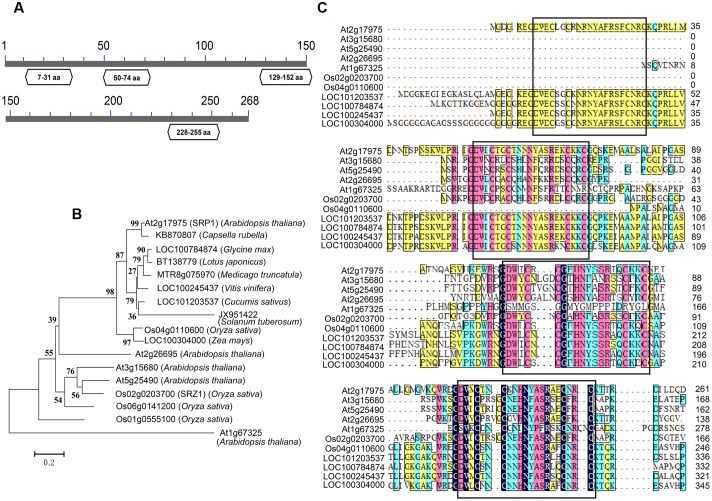
**Characterization of SRP1.** **(A)** The conserved domain analysis of SRP1. The conserved domains in SRP1 were identified in NCBI (http://www.ncbi.nlm.nih.gov). The zinc finger domains are shown in boxes with domain positions. The numbers represent the amino acids. **(B)** Phylogenetic analysis of C2C2 type zinc-finger proteins. Phylogenetic analysis of SRP1 and other plant C2C2 type zinc-finger proteins was performed using the neighbor-joining method wrapped in MEGA. Numbers at the nodes indicate bootstrap values (1000 replicates). **(C)** Sequence alignment of SRP1 and other plant C2C2-type zinc-finger proteins. The zinc finger motifs are boxed, respectively.

Previously we reported a stress repressive zinc finger protein SRZ1 in rice (Huang et al., 2008). In present study, we studied the function of SRP1, the homolog of SRZ1 in *Arabidopsis*. The *SRP1* (AT2G17975) gene possesses a translated region of 807-bp that encodes a protein containing 268 amino acids, with a calculated molecular weight of 30.24 kDa. On the basis of conserved domain analysis, four C2C2-type ZnF motifs were found in SRP1 and homologous to mammalian RanBP2-type ZnF domains (Nguyen et al., 2011; O’Connell et al., 2012; Vandevenne et al., 2014) (**Figure 1A**). The homologous proteins of SRP1 were found widely distributing among plant species, and they contained three or four C2C2-type ZnF motifs (**Figures 1B,C**). In *Arabidopsis*, four orthologs of *SRP1* were identified, but SRP1 was unique among those five homologous proteins for the extra ZnF motif in N-terminal.

### SRP1 Is Down-regulated by ABA and Abiotic Stress

**FIGURE 2:**
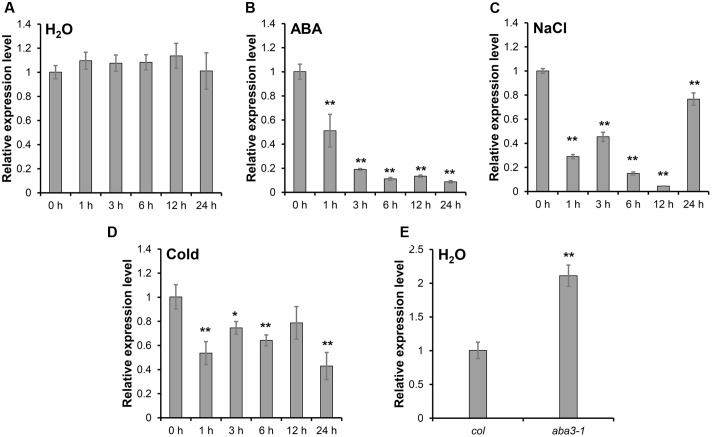
**The expression pattern of *SRP1* under abiotic stresses.** The seeds were germinated in the 1/2 MS medium. The 7-days-old seedlings were transferred into the control or stress conditions to analyze the expression pattern of *SRP1* by qRT-PCR. “^∗^”, “^∗∗^” represent significant differences relative to each control and *P*-value < 0.05 or *P*-value < 0.01, based on student’s *t*-test. Each value was the mean ± SD of three biological determinations. **(A)** The expression of *SRP1* in WT. **(B)** The expression of *SRP1* in WT treated with 10 μM ABA. **(C)** The expression of *SRP1* in WT treated with 100 mM NaCl. **(D)** The expression of *SRP1* in WT treated with cold stress (4 °C). **(E)** The expression of *SRP1* in *aba3-1*.

The expression of *SRP1* was analyzed in the WT seedlings treated with ABA, salt stress and cold stress. *SRP1* expression was down-regulated under the treatment of ABA within 24 h and gradually decreased along with the treatment time (**Figures 2A,B**). The expression of *SRP1* was also repressed by salt or cold stresses (**Figures 2C,D**). Compared with WT, the expression of *SRP1* in *aba3-1* was up-regulated (**Figure 2E**). The *aba3-1* is ABA deficient mutant, and the level of endogenous ABA in *aba3-1* is reduced as the conversion of ABA-aldehyde to ABA is blocked (Léon-Kloosterziel et al., 1996). These results indicate the expression of *SRP1* is repressed by abiotic stress and negatively regulated by ABA. It is of interest to investigate the involvement of *SRP1* in ABA signaling as it is significantly down-regulated by ABA (**Figure 2B**).

### SRP1 Modulates Seed Germination under ABA Treatment

**FIGURE 3:**
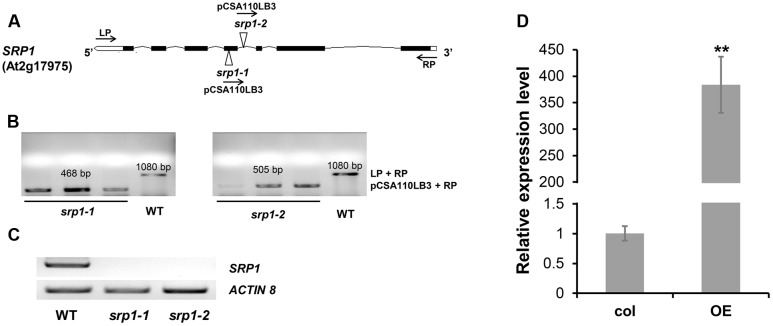
**Identification of *srp1* and *35S::SRP1*.** **(A)** Position of the T-DNA insertions in the *SRP1* genomic region. Black boxes and lines depict the exons and introns, respectively. White boxes depict the UTR regions and the Triangles depict the T-DNA insert location. The arrows depict the primers used in the genomic PCR identification**. (B)** The genomic PCR identification of T-DNA insertion. The homozygous mutants were verified by amplifying the overlapping region of T-DNA insertion and the gene. **(C)** The semi-quantitative RT-PCR analysis of the mutants. *Actin 8* was used as the internal control. **(D)** The qRT-PCR analysis of *35S::SRP1* (OE). “^∗∗^” represents significant difference relative to each control and *P*-value < 0.01, based on student’s *t*-test. Each value was the mean ± SD of three biological determinations.

The *SRP1* T-DNA insertion mutants *srp1-1* and *srp1-2* (**Figure 3A**) were verified via genomic PCR (**Figure 3B**) and semi-quantitative RT-PCR (**Figure 3C**). The *35S::SRP1* plants (T_4_ generation) were used in subsequent analysis. By qRT-PCR verification, it was found that *SRP1* was highly expressed in the *35S::SRP1* plant (**Figure 3D**).

**FIGURE 4:**
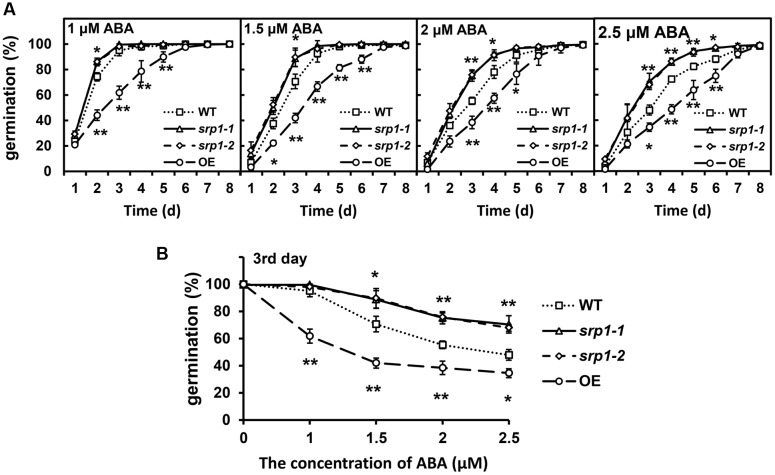
**Seed germination under ABA treatment.** **(A)** The germination of *srp1* mutants and OE lines in 1/2 MS medium supplemented with 1, 1.5, 2, and 2.5 μM ABA. The time indicates the days after vernalization. **(B)** The germination of *srp1* mutants and OE lines in 1/2 MS medium supplemented with different concentration of ABA on the 3^rd^ day after vernalization. “^∗^”, “^∗∗^” represent significant differences relative to each control and *P*-value < 0.05 or *P*-value < 0.01, based on student’s *t*-test. Each value was the mean ± SD of three biological determinations.

The seeds of the WT, *srp1-1. srp1-2* and *35S::SRP1* plants were germinated on 1/2 MS medium supplemented with or without ABA to analyze the sensitivity to ABA. The germination rates among WT, *srp1-1. srp1-2*, and *35S::SRP1* plants were similar on the medium under control condition (**Supplementary Figure 1A**). As shown in **Figure 4A**, the germination rates among WT, *srp1-1* and *srp1-2* were similar on the 1/2 MS medium supplemented with 1 μM ABA. However, the germination rates of *srp1-1* and *srp1-2* seeds were significantly higher than WT on the third day when 1.5 or 2 μM exogenous ABA was applied. The germination rates of mutant seeds with 2.5 μM ABA treatment were significantly higher than WT from the third to the sixth day. The germination rate of WT was reduced by 4.97, 29.36, 44.64, or 52.06%, respectively, on the third day when 1, 1.5, 2, or 2.5 μM exogenous ABA was applied. However, the germination rate of *srp1-1* was reduced by 0.05, 10.74, 24.38, or 29.33%, while *srp1-2* was reduced by 2.05, 10.29, 24.28, or 32.10%. The difference between the mutants and WT was increased with the ABA concentration (**Figure 4B**). In contrast, the *35S::SRP1* line exhibited significantly lower germination rate than WT under ABA treatment (**Figure 4A**).

### SRP1 Modulates Post-germinative Growth under ABA

**FIGURE 5:**
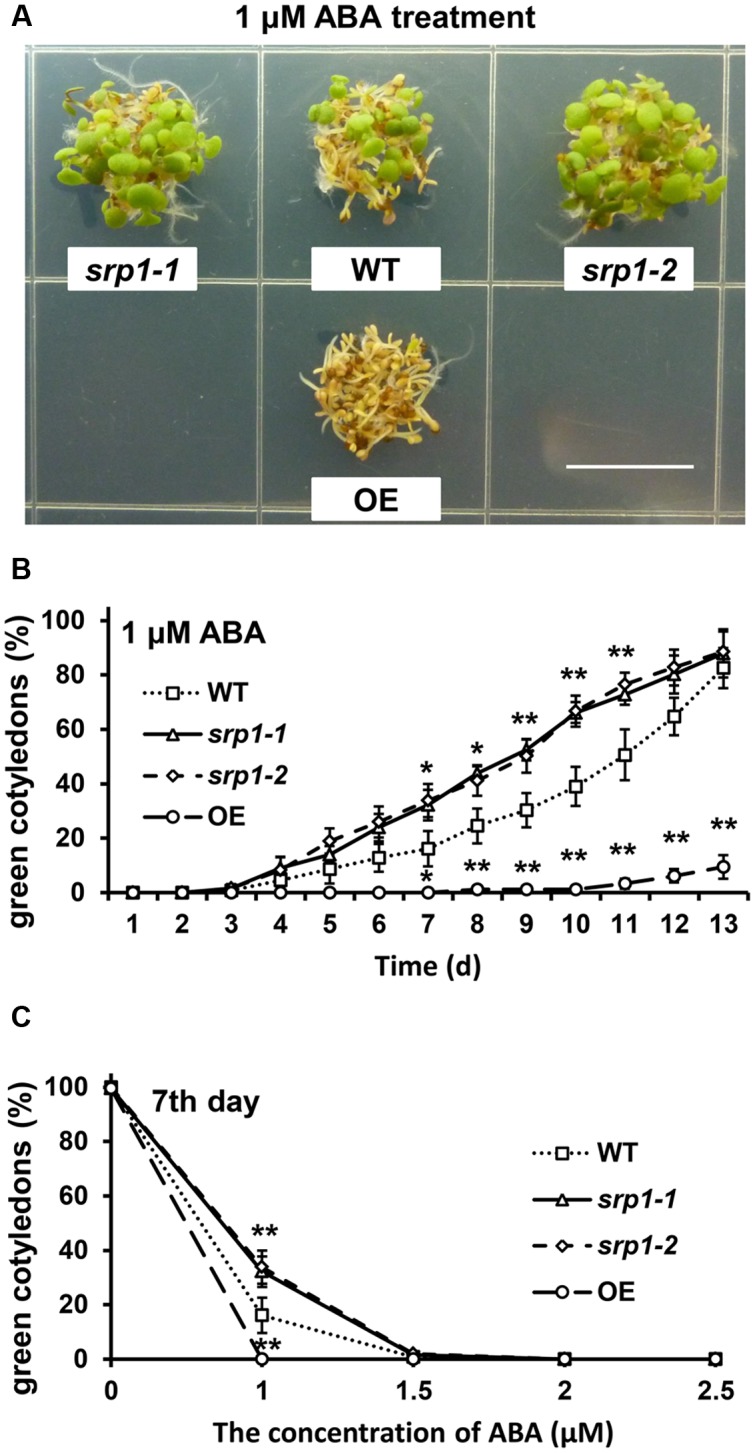
**Post-germinative growth under ABA treatment.** **(A)** The green cotyledons of *srp1* mutants and OE lines in 1/2 MS medium supplemented with 1 μM ABA. Scale = 1 cm. **(B)** The green cotyledon rate of *srp1* mutants and OE lines in 1/2 MS medium supplemented with 1 μM ABA. The time indicates the days after vernalization. **(C)** The green cotyledon rates of *srp1* mutants and OE lines in 1/2 MS medium supplemented with 1, 1.5, 2, and 2.5 μM ABA on the 7th day after vernalization. “^∗^”, “^∗∗^” represent significant differences relative to each control and *P*-value < 0.05 or *P*-value < 0.01, based on student’s *t*-test. Each value was the mean ±*SD* of three biological determinations.

The green cotyledon rates of the WT, *srp1-1. srp1-2*, and *35S::SRP1* plants were also analyzed to find out the sensitivity of *Arabidopsis* to ABA during the post-germinative growth stage. The green cotyledon rates among WT, *srp1-1. srp1-2*, and *35S::SRP1* plants were similar under control condition (**Supplementary Figure 1B**). Seven days after the incubation on the 1/2 MS medium supplemented with 1 μM ABA, the cotyledon greening efficiencies of *srp1-1* and *srp1-2* were significantly higher than WT, while *35S::SRP1* line was significantly lower (**Figure 5A**). The green cotyledon rates of *srp1-1* and *srp1-2* were significantly higher than WT from the seventh day to the eleventh day when 1 μM exogenous ABA was applied, and the difference between mutants and WT was extremely significant from the ninth day to the eleventh day (**Figure 5B**). The green cotyledon rates of mutants and WT treated with 1.5, 2, or 2.5 μM exogenous ABA were also analyzed but no significant difference was observed (**Figure 5C**). Moreover, the green cotyledon rates of mutants and WT were decreased along with the ABA concentration. In contrast, the *35S::SRP1* plants treated with 1 μM exogenous ABA exhibited significantly lower green cotyledon rate than WT from the seventh day after sowing (**Figure 5B**). These results indicated that the *srp1* mutants were more insensitive to exogenous ABA than the WT during the post-germinative growth, while the *35S::SRP1* plant was more sensitive.

### SRP1 Modulates Seed Germination and Post-germinative Growth under NaCl

**FIGURE 6:**
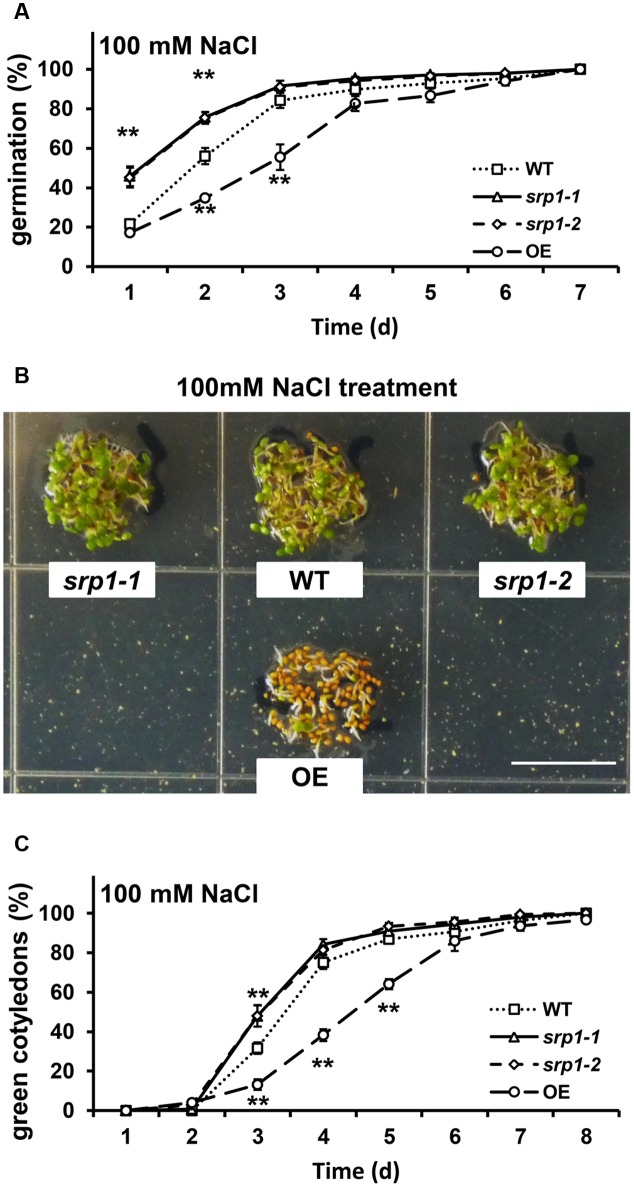
**Seed germination and post-germinative growth under NaCl treatment.** **(A)** The germination of *srp1* mutants and OE lines in 1/2 MS medium supplemented with 100 mM NaCl. **(B)** The green cotyledons of *srp1* mutants and OE lines in 1/2 MS medium supplemented with 100 mM NaCl. Scale = 1 cm. **(C)** The green cotyledon rate of *srp1* mutants and OE lines in 1/2 MS medium supplemented with 100 mM NaCl. The time indicates the days after vernalization. “^∗∗^” represents significant difference relative to each control and *P*-value < 0.01, based on student’s *t*-test. Each value was the mean ± SD of three biological determinations.

In the presence of 100 mM NaCl, the germination rates of *srp1-1* and *srp1-2* seeds were significantly higher than WT in the first two days (**Figure 6A**), and the cotyledon greening efficiencies of *srp1-1* and *srp1-2* were significantly higher than WT on the third day (**Figures 6B,C**). In contrast, the *35S::SRP1* line exhibited significantly lower germination and green cotyledon rate than WT under NaCl treatment (**Figure 6**).

### SRP1 Modulates the Expression of ABA Signaling-related Genes

**FIGURE 7:**
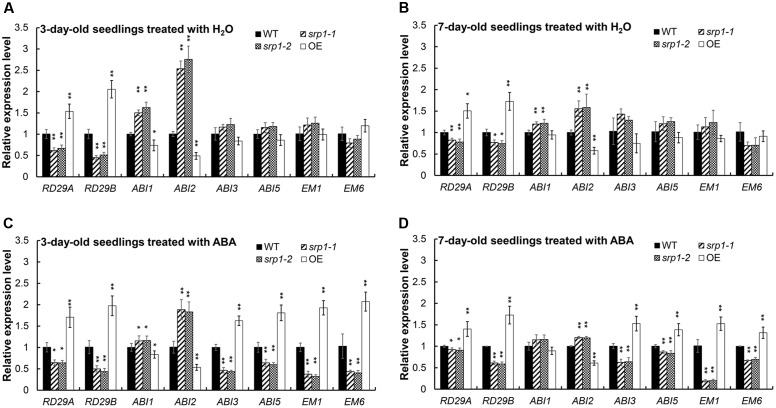
**The transcript abundances of the genes involved in ABA signaling.** **(A)** Transcript abundances of *RD29A, RD29B, ABI1, ABI2, ABI3, ABI5, EM1*, and *EM6* in 3-day-old seedlings grown in MS medium for 24 h were analyzed by qRT-PCR. **(A,B)** Transcript abundances of *RD29A, RD29B, ABI1, ABI2, ABI3, ABI5, EM1* and *EM6* in 7-day-old seedlings grown in MS medium for 24 h were analyzed by qRT-PCR. **(C)** Transcript abundances of *RD29A, RD29B, ABI1, ABI2, ABI3, ABI5, EM1*, and *EM6* in 3-day-old seedlings grown in MS medium containing 2.5 μM ABA for 24 h were analyzed by qRT-PCR. **(D)** Transcript abundances of *RD29A, RD29B, ABI1, ABI2, ABI3, ABI5, EM1*, and *EM6* in 7-day-old seedlings grown in MS medium containing 2.5 μM ABA for 24 h were analyzed by qRT-PCR. “^∗^”, “^∗∗^” represent significant differences relative to each control and *P*-value < 0.05 or *P*-value < 0.01, based on student’s *t*-test. Each value was the mean ± *SD* of three biological determinations.

The contents of endogenous ABA in *srp1* and WT were analyzed and we did not observe the marked differences among the mutant, *35S::SRP1* plant and WT (**Supplementary Figure 2**). The transcript abundances of ABA signaling and germination-related gene *RD29A, RD29B, ABA INSENSITIVE 1 (ABI1), ABA INSENSITIVE 2 (ABI2), ABA INSENSITIVE 3 (ABI3), ABA INSENSITIVE 5 (ABI5), LATE EMBRYOGENESIS ABUNDANT 1 (EM1). and LATE EMBRYOGENESIS ABUNDANT 1 (EM6)* were analyzed in the *srp1* mutants. As shown in **Figures 7A,B**, the transcript levels of *RD29A* and *RD29B* were repressed by *SRP1* knock-out under the control condition, but the transcriptions of *ABI1* and *ABI2* were enhanced. The transcript levels of *RD29A. RD29B. ABI3. ABI5. EM1*, and *EM6* were significantly less induced in *srp1* mutants than in WT under ABA treatment (**Figures 7C,D**). Interestingly, the transcript level of *ABI2* was significantly increased in the *srp1* mutants under control and ABA conditions, while that of *ABI1* was slightly increased under control condition. In addition, the expressions of those genes in *35S::SRP1* plant were opposite. These results indicate the involvement of *SRP1* in ABA signaling.

### SRP1 Possess the Dual Function in ABI2 3′UTR RNA Binding and Transcriptional Activation

**FIGURE 8:**
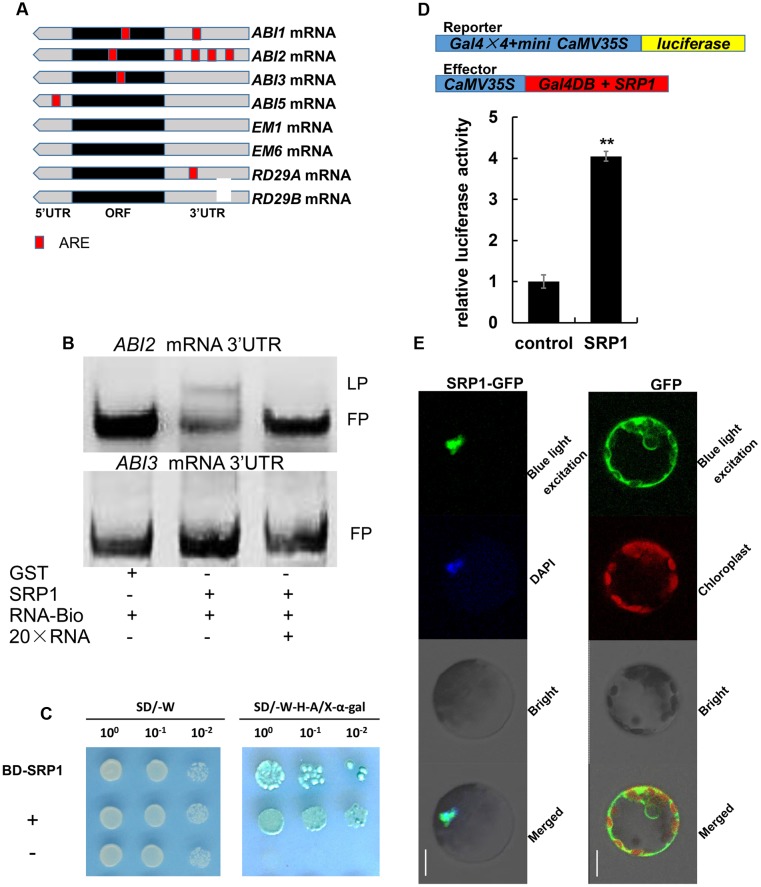
**The *ABI2* mRNA binding and transcriptional activity of SRP1.** **(A)** The core sequence of ARE element (AUUUA) in ABA signaling-related genes. The red boxes depict the location of AUUUA sequence. The gray and black boxes depict the UTR and ORF, respectively. **(B)** The RNA-EMSA analysis of SRP1 binding ability to *ABI2/3* 3′UTR. GST protein was used as the negative control. The RNA (*ABI2/3* 3′UTR) labeled with biotin was used as the probe. The unlabeled RNA (*ABI2/3* 3′UTR) was used as the competitor. LP, lagged probe. FP, free probe. **(C)** Transactivation analysis of SRP1 in the yeast GAL4 system. SRP1-pGBKT7 was transformed into yeast to analyze the transcriptional activity (BD-SRP1). The autonomously activated SRP1-pGBKT7 indicated the transcriptional activity of SRP1. The transformation of empty pGBKT7 was used as the negative control (–) while the co-transformation of pGBKT7-53 and pGADT7-T was used as the positive control (+). **(D)** Transactivation analysis of SRP1 in *Arabidopsis* protoplast system. 35S-Gal4DB was used as the negative control (control). The 35S-SRP1 was used as the effector to test transactivation activity of SRP1 (SRP1). Relative activity was normalized by the activity of *Renilla reniformis* luciferase. “^∗∗^” represents significant difference relative to each control and *P*-value < 0.01, based on student’s *t*-test. Each value was the mean ±*SD* of three biological determinations. **(E)** Subcellular localization of SRP1. The subcellular localization of SRP1 was analyzed in *Arabidopsis* leaf protoplasts. The green fluorescent protein SRP1-GFP fusion product was transiently expressed in *Arabidopsis* leaf protoplasts. The nucleus was dyed with DAPI.

It has been demonstrated that AU rich element (ARE) could be bound by RBP (Lai and Blackshear, 2001), and the core sequence of ARE is AUUUA. In this study, the distributions of AUUUA sequence in ABA signaling-related genes were analyzed. Interestingly, four AUUUA sequences were found in the 3′UTR of *ABI2* while zero or fewer AUUUA sequences were found in other genes (**Figure 8A**). As shown in **Figure 8B**, the biotin labeled *ABI2* 3′UTR mRNA lagged in the wells containing SRP1 protein. Moreover, the signals of lagging probes were reduced along with the addition of *ABI2* 3′UTR mRNA without labeling. By contrast, the biotin labeled *ABI3* 3′UTR mRNA did not lag in the wells containing SRP1 protein (**Figure 8B**). These results indicate the activity of SRP1 in binding to *ABI2* 3′UTR rather than *ABI3* 3′UTR. In addition, SRP1 was found with the transcriptional activation activity in yeast GAL4 system (**Figure 8C**). To further confirm the result, we examined the transcriptional activation activity of SRP1 in *Arabidopsis* protoplast system. As shown in **Figure 8D**, SRP1 could increase the luciferase activity by four-fold, indicating the transcriptional activation role of SRP1. The SRP1-green fluorescent protein (GFP) fusion protein was transiently expressed in *Arabidopsis* protoplasts. The SRP1 was revealed to be localized in nucleus of *Arabidopsis* protoplast cells (**Figure 8E**). The result suggested the function of SRP1 was performed in the nucleus.

### SRP1 Affects the Stability of mRNA Fused with ABI2 3′UTR

**FIGURE 9:**
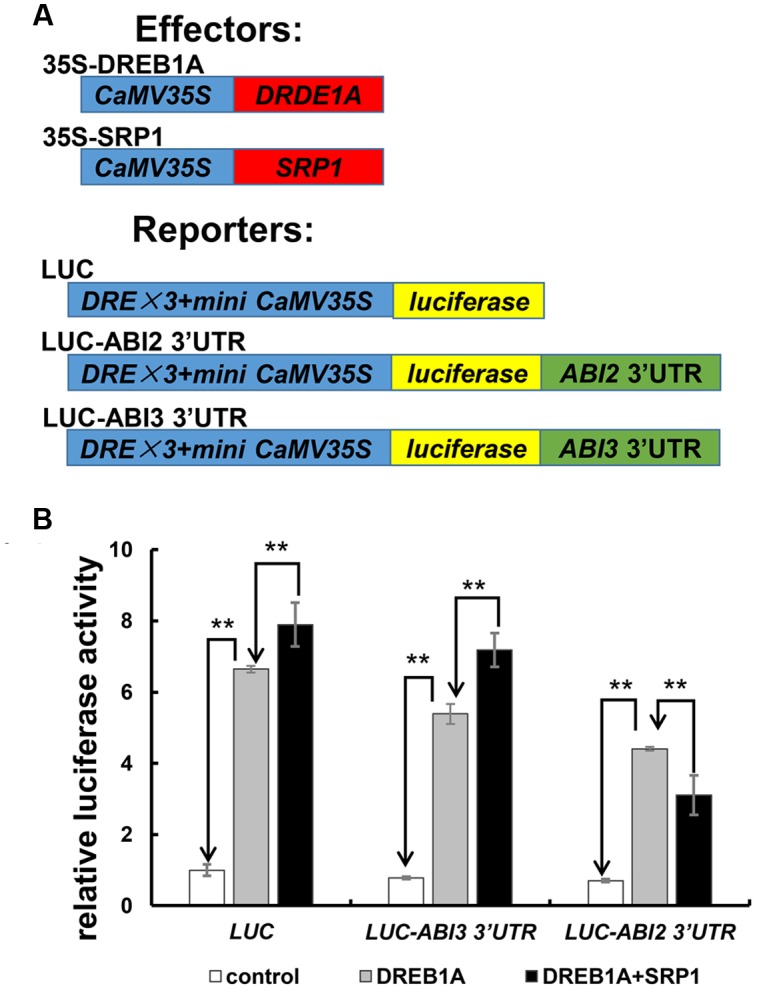
**The effect of SRP1 on the stability of mRNA with *ABI2* 3′UTR.** **(A)** Effectors and reporters used in transient expression analysis. 35S-DREB1A and 35S-SRP1 was used as the effectors. LUC effector was constructed by inserting three copies of DRE and a mini 35S into the upstream of the *Luciferase* in pGL3-basic vector. LUC-ABI2/ABI3 3′UTR effector was constructed by inserting the 3′UTR of ABI2/ABI3 into the downstream of *Luciferase* in LUC. **(B)** The effect of SRP1 on the activities of reporter vectors (LUC, LUC-*ABI2* 3′UTR and LUC-*ABI3* 3′UTR). The empty PA7 vector was used as the negative control (control). As SRP1 possessed the transactivation activity, 35S-DREB1A effector was used as the positive control to enhance the expression of reporter (DREB1A). The 35S-DREB1A, 35S-SRP1 and reporters were co-transfected into *Arabidopsis* protoplasts to test the effect of SRP1 on the stability of mRNA (DREB1A + SRP1). Relative activity was normalized by the activity of *Renilla reniformis* luciferase. “^∗∗^” represents significant difference relative to each control and *P*-value < 0.01, based on student’s *t*-test. Each value was the mean ±*SD* of three biological determinations.

The effect of SRP1 on *ABI2* 3′UTR-containing mRNA stability was examined in *Arabidopsis* protoplast cells to confirm whether the binding of SRP1 to *ABI2* 3′UTR mediated mRNA silencing (**Figure 9**). In this experiment, two reporter vectors, LUC-*ABI2* 3′UTR and LUC-*ABI3* 3′UTR were constructed by inserting the *ABI2* or *ABI3* 3′UTR into the downstream of luciferase coding sequence in the control reporter LUC (**Figure 9A**). All of the reporter vectors were trans-activated by dehydration-responsive element-binding protein 1A (DREB1A), but the luciferase activity was reduced if an additional *ABI2* 3′UTR was fused with luciferase gene (**Figure 9B**). Importantly, the luciferase activity of LUC-*ABI2* 3′UTR was reduced by the addition of SRP1 while the luciferase activities of LUC and LUC-*ABI3* 3′UTR were enhanced (**Figure 9B**). Considering the transcriptional activity of SRP1 observed in yeast and protoplast systems, all three of these vectors were supposed to be enhanced. Hence, the decreased luciferase activity of LUC-*ABI2* 3′UTR confirmed the role of SRP1 in reducing the stability of mRNA with 3′UTR of *ABI2*.

## Discussion

The post-transcriptional regulation in ABA signaling is critical but not fully known. In present study, a novel RBP was found regulating ABA signaling at post-transcriptional level. Several RBPs containing CCCH-type zinc finger motif have been characterized, such as atRZ-1a, OsTZF1 and AtTZF1 (Kim et al., 2007; Ambrosone et al., 2012; Qu et al., 2014). The CCCH-type ZnF motif is required to achieve high-affinity RNA-binding (Qu et al., 2014). SRP1 contains four C2C2-type ZnF motifs, and it has been poorly studied in plant. The mammal protein RanBP2 containing the similar motif has been reported to recognize and bind to the ssRNA (Nguyen et al., 2011). Unlike RanBP2 in mammals, SRP1 only contains four zinc finger motifs while RanBP2 is a big protein containing multiple functional domains besides zinc finger motifs. Other similar zinc finger motif containing proteins in mammal, such as ZnF Ran binding domain-containing protein 2 (ZRANB2) and RNA-binding motif 5 (RBM5), are reported to have the ssRNA-binding activity (O’Connell et al., 2012; Vandevenne et al., 2014). The database searching indicates that SRP1 is plant-specific and widely distributed in plant species, implying they may play a specific role in plants through the C2C2-type ZnF motifs.

Many RBPs have been shown with the involvements in abiotic stress (Ambrosone et al., 2012). The mRNA expression of a glycine-rich RBP atRZ1-a is down-regulated by ABA (Kim et al., 2005), and the expression of RRM containing RBP *ARP1* is also down-regulated by ABA (Jung et al., 2013). With the treatment of ABA, transgenic *Arabidopsis* plants overexpressing *atRZ-1a* display retarded germination and seedling growth compared with the WT plants while *atrz-1a* mutant germinates earlier and grows faster than the WT plants (Kim et al., 2007). Similarly, the expression of *SRP1* was down-regulated by ABA and abiotic stress. The *srp1* knock-out mutants showed ABA insensitivity, while *35S::SRP1* plant showed ABA hypersensitivity during the seed germination and post-germinative growth stages (**Figures 4** and **5**). These results support the notion that *SRP1* is a positive component of ABA-triggered responses in seed germination and seedling growth.

ABI proteins play important roles in ABA signaling during the germination. *ABI1. ABI2. ABI3*, and *ABI5* are induced by ABA, and the knock-out mutants of them are insensitive to ABA during the germination stage (Suzuki et al., 2003). With the treatment of ABA, these *ABI* genes were differentially regulated in *srp1* knock-out mutants in comparison with WT. Among them, *ABI3* and *ABI5* were significantly less induced by ABA in the mutants than WT. In contrast, *ABI1* was slightly more induced by ABA in the mutants than WT, while the expression of *ABI2* was significantly increased. Among these proteins, ABI3 is a B3 domain transcription factor playing an important role in inhibiting the germination under ABA treatment (Rohde et al., 2000). ABI1 and ABI2 are protein phosphatase 2C (PP2C) proteins negatively regulating ABA response (Schweighofer et al., 2004; Hubbard et al., 2010), and the induction of them by ABA is feedback inhibited by ABI3 (Suzuki et al., 2003). It seems that, the enhanced expression of *ABI1* and *ABI2* in *srp1* negatively regulates the seed sensitivity to ABA. The basic leucine zipper (bZIP)-type transcription factor ABI5, activated by ABI3, is an positive regulator in ABA signaling (Nakabayashi et al., 2005). Moreover, the expressions of *EM1. EM6. RD29A* and *RD29B* were less induced in the *srp1* mutants. Previous studies have revealed that *RD29A* and *RD29B* are up-regulated by ABA and ABI3 (Nakashima et al., 2006). ABI5 induces expressions of *EM1* and *EM6* which can inhibit the embryo development (Finkelstein and Lynch, 2000). These results suggest that the deficiency of *SRP1* decreases the seed sensitivity to ABA by inhibiting the expressions of *ABI3* and *ABI5*. Subsequently, *EM1* and *EM6* expression levels are decreased and the germination is delayed.

ABI2 plays important roles in both ABA and salt stress responses. The *abi2-1* exhibits increased tolerance to salt shock and ABA insensitivity by disrupting the interaction between SOS2 and ABI2 (Ohta et al., 2003). *HDA6* mutant, *axe1-5*, is hypersensitive to ABA and salt stress, and the expression of *ABI2* in that is decreased while treated with ABA or salt stress (Chen et al., 2010). Contrary to the phenotype of *axe1-5* under ABA, the *srp1* mutants showed reduced salt sensitivity during the seed germination and post-germinative growth stages (**Figure 6**), which was most probably due to the increased transcript level of *ABI2*.

Post-transcriptional regulation involves mRNA transport, localization, stability or translation through the interactions between RBPs and mRNAs (Tam et al., 2010; Qu et al., 2014). A well-documented example is AU rich element (ARE)-interacting protein hTTP, which can bind to the 3′UTR of mRNAs encoding regulators involved in innate immune responses and promote the degradation of them (Lai and Blackshear, 2001; Barreau et al., 2005). The 3′UTR of *ABI2* mRNA contains four core sequences of ARE (AUUUA), while no AUUUA sequence is present in the 3′UTR of *ABI3.* In accordance with this, SRP1 could interact with 3′UTR of *ABI2* rather than 3′UTR of *ABI3*. Correspondingly, SRP1 could cause the instability of the mRNA fused with *ABI2* 3′UTR rather than *ABI3* 3′UTR. These evidences support the hypothesis that SRP1 functions as an RBP triggering the instability of *ABI2* mRNA in ABA signaling by binding to its 3′UTR. Unlike the pre-mRNA splicing related CBP20 and RZ-1, SRP1 possesses function by regulating the stability of mRNA.

SRP1 was localized in the nucleus of *Arabidopsis*, and the function of it most probably performed there. Several RNase proteins are localized in same organelle, such as RNase III DICER-Like1 (DCL1), RNase D, RNase P, RNase Z, etc (Dubrovsky et al., 2003; Glazov et al., 2003; Song et al., 2007; Gobert et al., 2013). Those RNase proteins can cleave the special RNA sequences and play important roles in processing of RNA. DCL1 is essential for the accumulation of microRNA (Song et al., 2007). RNase D is a 3′ to 5′ exonuclease required for the processing of RNA (Glazov et al., 2003). RNase P plays the critical role in removing 5′-leader sequences from tRNA precursors (Gobert et al., 2013). RNase Z is important for the generation of mature tRNA 3′ end (Dubrovsky et al., 2003). The further study is necessary to reveal whether SRP1 possesses the cleaving function as nucleus-localized RNase or cooperates with them.

**FIGURE 10:**
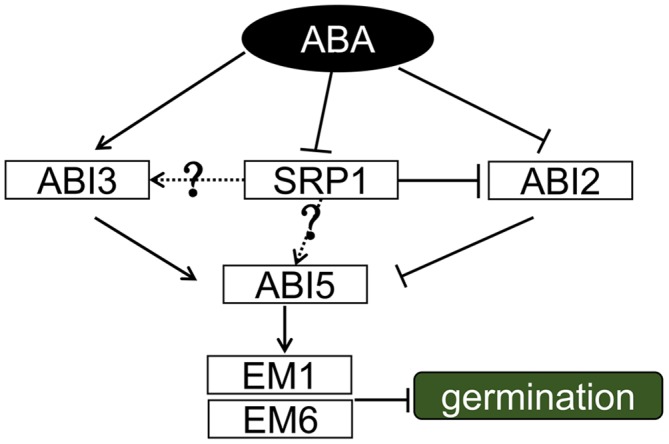
**A proposed working model for SRP1.** During ABA signaling, ABA induced expression of *ABI3/5* and inhibited the activities of *ABI2. ABI2* was post-transcriptional modulated by SRP1, and the degradation of *ABI2* mRNA was repressed when *SRP1* is down-regulated under ABA. *ABI3* and *ABI5* were down-regulated in *srp1*. It was assumed that the down-regulation of *SRP1* under ABA results in the suppression of *ABI3* and *ABI5*, but more evidence was necessary to demonstrate it. The solid lines depict the relationships proved in this or previous studies, and the dotted lines and question marks depict the unproved relationships.

Our work demonstrates that SRP1 is an RBP functioning in ABA signaling and exerts its roles by modulating the levels of *ABI* genes (**Figure 10**). Among these genes, *ABI2* is directly post-transcriptionally modulated by SRP1. More studies are required to reveal the relationships between SRP1 and other *ABI* genes, e.g., *ABI3* and *ABI5*. The transcriptional activation activity of SRP1 implies the dual roles of SRP1 in transcriptional and post-transcriptional regulations on target genes. Conclusively, the present study identified a novel plant-specific RNA-binding protein involving ABA signaling by post-transcriptionally repressing *ABI2*.

## Author Contributions

JH planned the project. JX, YC, RM, XY, HF, XH, EX, and LQ performed the experiments. JX, HZ, and JH analyzed the data. JX and JH wrote the article.

## Conflict of Interest Statement

The authors declare that the research was conducted in the absence of any commercial or financial relationships that could be construed as a potential conflict of interest.

## Footnotes

1 www.arabidopsis.org
