# Supplementary material for: A Novel RNA-Binding Protein Involves ABA Signaling by Post-transcriptionally Repressing ABI2
Source: Front Plant Sci. 2017 Jan 24;8:24. doi: 10.3389/fpls.2017.00024 (PMC5258706; doi:10.3389/fpls.2017.00024)
Supplement: Supplementary file 1 [file Data_Sheet_1.docx]

Supplementary Material

A novel RNA-binding protein regulates ABA signaling by targeting *ABI2*

Jianwen Xu^1, 3^, Yihan Chen^1,2^, Rong Mu^1,2^, Xi Yuan^1,2^, Huimin Fang^1,2^, Xi Huang^1,2^, Enshun Xu^1,2^, Hongsheng Zhang^1,2^ and Ji Huang^1,2*^

*** Correspondence:** Ji Huang: huangji@njau.edu.cn

# Supplementary Figures and Tables

## Supplementary Figures


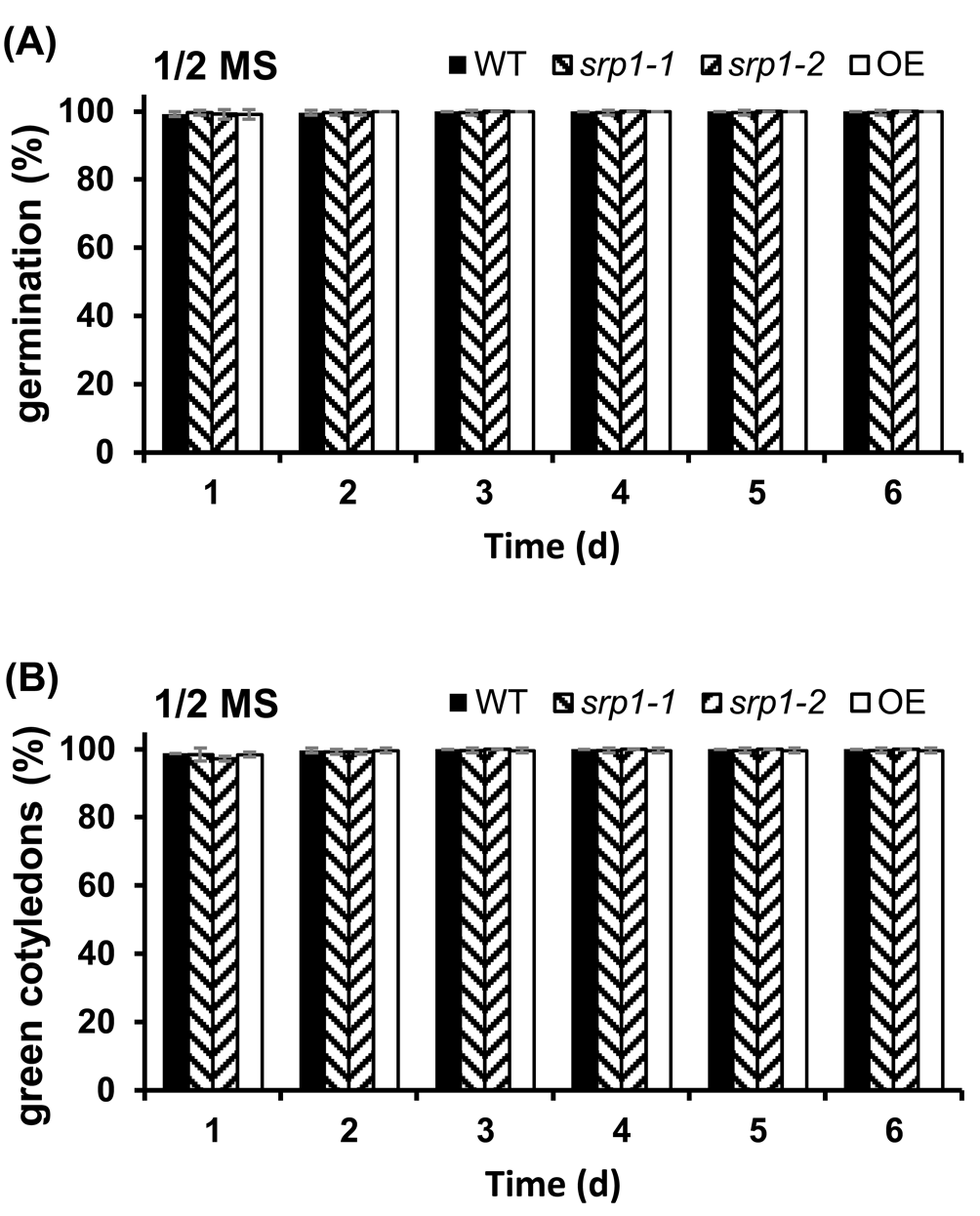


**Supplementary Figure 1.** Seed germination and post-germinative growth under normal conditions. **(A)** The germination of *srp1* and *35S::SRP1* plant (OE) in 1/2 MS medium. **(B)** The green cotyledon rates of *srp1* and OE in 1/2 MS medium.

**
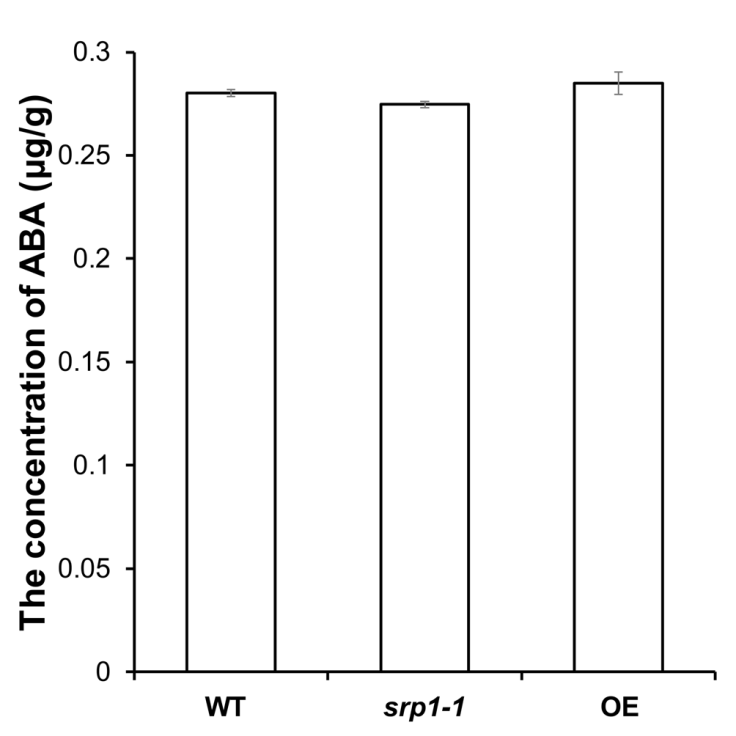
**

**Supplement Figure 2.** Measurement of ABA contents in srp1 and WT.

## Supplementary Figures and Tables

**Supplementary Table 1.** The primers used in the experiment.

| **primer** | **sequence (5'-3')** |
| --- | --- |
| **primers for PCR** | |
| LP | TATATGGAATTTGCTGGTGGG |
| RP | TATTGCACTCTGCTCGTGATG |
| pCSA110LB | TATATGGAATTTGCTGGTGGG |
| hygromycin -F | GCTGTTATGCGGCCATTGTC |
| hygromycin -R | GACGTCTGTCGAGAAGTTTG |
| SRZ5-F | CGCCGGAGACTGGAGTTT |
| SRZ5-R | AGCCATGTCTCACTAGATTAAG |
| ACTIN8-F | ATGAAGATTAAGGTCGTGGC |
| ACTIN8-R | TCCGAGTTTGAAGAGGCTAC |
| SRZ5-GST-F | ACGCGTCGACGTATGGTGAGAAAGAGAAGAAC |
| SRZ5-GST-R | CCCAAGCTTGGCAGTAGAACATGACACGCTTC |
| ABI2-3U-probe-F | TAATACGACTCACTATAGGAAAAGAAGGTT |
| ABI2-3U-probe-R | GAAACATCAAATAATAACACTAAATATTTA |
| ABI3-3U-probe-F | TAATACGACTCACTATAGGAAAACAGAGAC |
| ABI3-3U-probe-R | AAAGAGTTATAAAAGAACCGACTCGACAAG |
| SRZ5-GFP-F | AACAGGTACCATGGGAGACGGAAGAG |
| SRZ5-GFP-R | TCAGATCTCTCAAGATTGTTCTGTT |
| **primers for qRT-PCR** | |
| ACTIN7-F | AGGGAGAAGATGACTCAGATC |
| ACTIN7-R | GTGTGAGACACA CCATCACC |
| ABI1-F | CATGTCGAGATCCATTGGCGATAG |
| ABI1-R | ACTCTCTTCACAGCCGTCACTTC |
| ABI2-F | CTCGCAATGTCAAGATCCATTGGC |
| ABI2-R | TTACTCGCCGCACTGAAGTCAC |
| ABI3-F | GAAGCAAAGCGACGTGGGTAAC |
| ABI3-R | AACCTGTAGCGCATGTTCCAAAC |
| ABI5-F | AGTCTGCTGCTAGATCTAGAG |
| ABI5-R | TGTTGCTTCCTCTTCCTCTCC |
| RD29A-F | TGGACAAAGCAATGAGCATGAGC |
| RD29A-R | AGGTTTACCTGTTACGCCTGGTG |
| RD29B-F | ACTGATCCCACGCATAAAGGTG |
| RD29B-R | CTCGTCGGAAAGTCTTCTTCGC |
| Em1-F | TTGCTGAAGGAAGGAGCAAGGG |
| Em1-R | TCCACCTTTGTGACCGATCTCC |
| Em6-f | TCAACAGCATCTCGCTGAAGGG |
| Em6-R | TCCGGTGCTAAGACCACCTTTG |
| SRP1-F | CATTCTCTAACGCTACAA |
| SRP1-R | CTTCTGATGCTAATCTCTT |

**Supplementary Table 2.** The sequences of *ABI2* and *ABI3* 3’UTRs.

| **gene** | **sequences** |
| --- | --- |
| *ABI2* 3’UTR | TAATACGACTCACTATAGGAAAAGAAGGTTTGGAAGAAAAGTGAAAAAAAAAGTTTTGATGGTGGGTAAAAATTCTCTTTAGTGAAAAAAGAAAGATAAAACAACAGGTAATAATTACATTGTAATATTAATTTCCTGCTTAAATTTGTTATTTACTTTCTTAGTCTTCCCACCATTATCTTTTTATATTTATTTTACTAACATGATATATATAATGTACATGGGAATAGTGTAACTTCCAAACAAAAAGCCCCCTTGTATTTTTTTTTATCTTGGGGTTAGTTGTAATAACACTACATTCGTCTACGTAAATATTTAGTGTTATTATTTGATGTTT |
| *ABI3* 3’UTR | TGAAAAACAGAGACAAAAAGAAACAATATAAATATTATTATGTACCAAATAAGAAAGAGGGCAAAAGGAAAAAATGGCAGCGTACCCGAGTGTGCCACTTCTCGTGCATGCATGGGATCTTGAAGACAAATGGAGGGTCATGATTAAAGCTGTTTGGTCGGGGTCCGGGTTTTTACTCCATTTTTTGCTTTTTCTTGTCGAGTCGGTTCTTTTATAACTCTTTACTCTTTTTACCTTCAGGATATTGTAGAGATGATTAATTCTGGAAATGGTGTTTGTGTTATAT |
